# Psychometric validation of the organizational climate construct in high-complexity hospitals in Chile and Ecuador

**DOI:** 10.3389/fpsyg.2026.1813699

**Published:** 2026-06-19

**Authors:** Miguel A. Bustamante-Ubilla, Mauricio Carvache-Franco, Orly Carvache-Franco, María del Carmen Lapo, Wilmer Carvache-Franco

**Affiliations:** 1 Facultad de Economía y Negocios, Universidad de Talca, Talca, Chile; 2 Sistema de Posgrado, Universidad Católica de Santiago de Guayaquil, Guayaquil, Ecuador; 3 Universidad Bolivariana del Ecuador, Durán, Ecuador; 4 Graduate School of Business, Universidad ESAN, Lima, Peru; 5 Universidad Espíritu Santo, Samborondón, Ecuador; 6 Universidad Católica de Santiago de Guayaquil, Guayaquil, Ecuador; 7 Facultad de Ciencias Sociales y Humanísticas, Escuela Superior Politécnica del Litoral (ESPOL), Guayaquil, Ecuador

**Keywords:** confirmatory factor analysis, exploratory factor analysis, high-complexity hospitals, organizational climate, psychometric validation, public health

## Abstract

This study aimed to psychometrically validate the construct of organizational climate in high-complexity hospitals in Chile and Ecuador. In both Chile and Ecuador, healthcare institutions, whether public or private, are organized according to levels of complexity. Consequently, considering the coverage and impact of services, the study focused on high-complexity institutions. A descriptive and correlational non-experimental study was conducted, applying exploratory factor analysis and structural equation modeling to confirm the underlying factor structure. Procedurally, a 27-item Likert-scale questionnaire was administered to a sample of 1,222 participants selected through convenience sampling, segmented into two parts: 639 from Chile and 583 from Ecuador. The findings confirmed adequate psychometric properties of the instrument in both contexts, including satisfactory reliability, factorial validity, and structural consistency across the analyzed hospital samples. In Chile, these factors are: 1. Support for management and work environment, 2. Tensions and conflicts, and 3. Support for intrapreneurship. In Ecuador, the factors are: 1. Relationships, friendship, and growth, 2. Collaboration, respectful treatment, support, and trust, and 3. Support for intrapreneurship. Finally, it is concluded that the first factor assumes an active, independent role; the second factor occupies a mediating role; and the third is established as endogenous or dependent. These three factors, in each context, establish relationships and mutual influences, forming an organizational climate construct duly confirmed in their respective realities. In summary, and in accordance with the findings of this study, a wide range of possibilities opens up for new scientific research in the health sector. It is suggested that this research could focus on primary care segments due to their coverage, dispersion, and diversity of services, as well as potentially conducting comparative studies of organizational climate in public or private institutions located in Latin American countries.

## 1 Introduction

Health is recognized as a fundamental human right by the World Health Organization (World Health Organization, 2019), a principle intended to ensure equitable access to High-complexity hospitals. In South America, efforts to achieve universal health coverage have advanced; however, in Ecuador and Chile, the two countries examined in this study, significant gaps in population well-being remain World Health Statistics (2021).

Addressing these deficiencies requires not only adequate health infrastructure, but also effective organizational management that allows for the management of perceptions, such as those of the organizational climate within highly complex health institutions (Angeles-Cabrera et al., 2026).

In this context, organizational climate has become established as a relevant management tool in psychology and human resource management (Morales et al., 2026) that refers to the perceptions that employees have about the environmental context in which they work, since they influence their behavior and job satisfaction (Nieto et al., 2026), opening the space to the need to carry out psychometric studies of items, factors and scales (Ale, 2026; Escalante, 2026) that allow a better understanding of the climate in complex organizations (Hernandez and Diaz, 2026).

Organizational climate has been described as a set of workplace characteristics that drive employee performance (Hernandez and Diaz, 2026) and productivity (Vasquez et al., 2026). However, a more subjective perspective has been incorporated into its analysis, suggesting the need for psychometric studies (Hernandez and Diaz, 2026). This perspective argues that the most important factor is how each individual develops their soft skills (Ale, 2026), and that, based on this, del Lapo-Maza María and Bustamante-Ubilla Miguel (2018) demonstrated that these personal perceptions directly affect workplace dynamics, specifically the quality of relationships with superiors and the frequency of prosocial behaviors derived from the social capital generated (Jiménez et al., 2026). Therefore, evaluating these perceptions using psychometric methods would allow for better management of human talent and the required communication policies (Angeles-Cabrera et al., 2026), as they significantly influence outcomes (Angeles-Cabrera et al., 2026). Thus, some studies have analyzed organizational climate in its various dimensions, including reciprocity and participation (Bustamante-Ubilla et al., 2018), which influence management practices, trust, and employee satisfaction (Armijos and Núñez, 2020).

In the healthcare sector, organizational climate is especially relevant given the complexity of the services provided and the demands placed on professionals (Ale, 2026). In this context, the climate is shaped by a combination of characteristics specific to the sector and its internal environmental factors (Angeles-Cabrera et al., 2026), forming particularly relevant factorial structures that should be studied psychometrically (Jiménez et al., 2026), considering the challenges of managing highly complex resources and, through them, ensuring equitable access to quality services (Morales et al., 2026; Nieto et al., 2026).

Thus, both nationally and internationally, various studies have documented the impact of organizational climate through psychometric studies (Rangel-Baca and Pacheco-Paz, 2026) focused on staff performance in high-complexity hospitals (Angeles-Cabrera et al., 2026), as well as addressing issues such as timeliness of care, social empowerment, and job satisfaction (Nieto et al., 2026). Consequently, it is vital for hospitals and healthcare providers at their various levels of complexity to promote research that seeks better talent management and improved relationships (Ale, 2026), demonstrating that understanding the internal work environment is fundamental for the continuous improvement of organizations (Alvear, 2026).

In general, studies on organizational climate analyze communication policies (Angeles-Cabrera et al., 2026), human resource management (Angeles-Cabrera et al., 2026), and social reciprocity behaviors (Bustamante-Ubilla et al., 2018) through specific studies. Others analyze organizational climate in relation to job satisfaction (Nieto et al., 2026), management practices (Armijos and Núñez, 2020), and job performance (Angeles-Cabrera et al., 2026), which opens the door to psychometric research that explores the underlying latent structures of organizational climate perceptions (Jiménez et al., 2026).

Consequently, and in accordance with the aforementioned, hospital subsystems generate characterizable environments, giving rise to factors that, with improved management, would allow for the appropriate resolution of potential tensions arising from the resulting organizational climates (Ale, 2026; Alvear, 2026).

For this reason, this study focuses on comparing the latent variables of organizational climate that naturally arise from the complexity of healthcare services (Ale, 2026) in two Latin American countries, Chile and Ecuador (Angeles-Cabrera et al., 2026). Therefore, it proposes to measure and compare the organizational climates of both countries using psychometric methods, as they face similar challenges (Morales et al., 2026; Nieto et al., 2026) and, consequently, may present comparable factorial components.

Although organizational climate has been widely studied in healthcare institutions, fewer studies have focused on the psychometric validation of organizational climate instruments in high-complexity hospital settings across different Latin American contexts. Existing research has mainly examined organizational climate outcomes rather than validating the factorial structures underlying these constructs. Consequently, there remains a need for comparative psychometric evidence capable of confirming whether organizational climate dimensions maintain structural consistency across distinct healthcare realities such as Chile and Ecuador.

Based on the space that the literature leaves available for new studies, the present work assumes the objective of psychometrically validating the Validated Organizational Climate Questionnaire (CCOV) proposed by Bustamante and Álvarez (2019) by contrasting the results of two high complexity hospital samples that come from two different South American realities such as Chile and Ecuador.

## 2 Methodology

The study was conducted in accordance with the guidelines of the Declaration of Helsinki and was approved by the Ethics Committee of the Escuela Superior Politécnica del Litoral (ESPOL), protocol code CERT-PI-CEIE-003-2023, on September 11, 2023. Informed consent was given in writing before the respondents filled out the questionnaire. As part of the ethical procedures, informed consent was included in the questionnaire used for data collection, which was previously accepted in writing by the participants.

This cross-sectional, correlational study was conducted in two countries using sequential analytical stages (Hernández-Sampieri and Mendoza, 2018). The first stage consisted of a descriptive analysis of the demographic characteristics of the studied populations. The second stage included an exploratory factor analysis applied to segmented databases, as has been demonstrated in similar studies (Bustamante et al., 2022). Finally, a confirmatory analysis was performed using structural equation modeling, a widely used approach for studies of this level (García et al., 2026; Escalante, 2026; Hernandez and Diaz, 2026), specifically to identify latent structures and test the psychometric validation hypotheses. This will allow for the synthesis of an organizational climate instrument validated in high-complexity hospitals in Ecuador and Chile.

### 2.1 Contextualization of the study

In Ecuador, the study was conducted in the province of Guayas, which, according to the National Institute of Statistics of Ecuador (INEC), had a population of 4,387,434 in 2020, representing 25.06% of the national total of 17,510,643 (MTOP, 2020). By 2024, Guayas had 672 health facilities, accounting for 16.20% of the national total and comprising both public and private institutions. Nationwide, morbidity consultations reached 38.8 million in 2019, of which 83.28% occurred in the public sector and 16.72% in the private sector, including both for-profit and non-profit facilities. In Guayas, data from 2024 showed a decrease in morbidity and mortality rates. However, these risks vary widely across the country and may constitute specific occupational hazards for healthcare workers (Gómez-García et al., 2024).

In Chile, the study was conducted in the Maule Region, which in 2024 had a population of 1,123,008 according to the national census (Maule Region Census, 2024) and the highest proportion of rural residents (32.9%).

Overall, 38% of the population is employed; however, 51.7% lack workers’ compensation insurance coverage. At the regional level, the availability of professional High-complexity hospitals per 1,000 inhabitants remains relatively low, with 6.9 h of dental services and 25 h of medical services (INEC, 2019). The Preventive Medicine Index increased by 61% between 1999 and 2003, though notable disparities were observed across the region’s 30 municipalities. Nevertheless, 67% of the eligible population did not have access to preventive services (Maule, 2021; Censo Región del Maule, 2014).

According to updated data from the Department of Statistics and Health Information (DEIS) of the Ministry of Health and the National System of Municipal Information (SINIM), as of January 12, 2024, the Maule Region had 24 Community Family Health Centers (CECOSF), 14 dialysis centers, 47 Family Health Centers (CESFAM), and 31 private Medium-complexity health centers to collectively support 31 public hospitals in the region Maule, (2024).

### 2.2 Design

A non-experimental correlational study was conducted (Hernández-Sampieri and Mendoza, 2018) using exploratory factor analysis to examine the underlying factor structure of the constructs studied (Jiménez et al., 2026). Subsequently, confirmatory factor analyses were performed to corroborate the identified dimensions (Ramírez, 2026; Rangel-Baca and Pacheco-Paz, 2026), and structural equation modeling was used to establish relationships between the variables (Jiménez et al., 2026). Demographic information was also collected from the sample, including gender (women and men), area of residence (urban or rural), and age (Hernández-Sampieri and Mendoza, 2018).

### 2.3 Sample size and sampling frame

The sample was selected using a two-stage sampling method (Hernández-Sampieri and Mendoza, 2018). In the first stage, participants were grouped into predefined strata based on their institutional affiliation. In the second stage, chain sampling was applied within each group (Zavaleta-de et al., 2020). The sampling frame (Hernández-Sampieri and Mendoza, 2018) was defined as the population of healthcare workers employed in high-complexity hospitals. Initial contact with participants was based on official information provided by the participating hospitals in each country. To ensure adequate statistical power (Secaira et al., 2026), a minimum sample size criterion was applied using the n/p (participants/items) rule, which recommends ratios of 5:1 to 10:1, requiring 5 to 10 participants per variable and a total sample size of at least 100 participants (Escalante, 2026).

The final sample comprised 1,222 participants, including 639 respondents from Chile and 583 from Ecuador. Data were collected using a questionnaire designed to capture perceptions on a five-point Likert-type scale (Hernández-Sampieri and Mendoza, 2018).

### 2.4 Inclusion and exclusion criteria

The inclusion criteria, consistent with previous studies, required that participants be working at their respective high-complexity healthcare centers (Nieto et al., 2026). Individuals on authorized leave or not employed at these centers at the time of data collection were excluded (Hernández-Sampieri and Mendoza, 2018). Access to participants was achieved through convenience sampling with chain recruitment (Iglesias-Armenteros et al., 2020), which allowed recruitment until the required sample size was reached (Escalante, 2026; García et al., 2026).

### 2.5 Survey team

A field team was formed, comprised of trained interviewers working under the coordination of the research team and a coordinator with direct links to the health centers (Hernández-Sampieri and Mendoza, 2018). The team included university graduates, professionals, and technicians trained in the research topics (Escalante, 2026; García et al., 2026). The interviewers were responsible for data collection, while an independent team of data entry operators managed the construction of the database (Pomachagua et al., 2026).

### 2.6 Interviewer training and procedures

The interviewers were trained by the researchers to ensure the standardized administration of the instrument (Hernández-Sampieri and Mendoza, 2018). Subsequently, a pilot application of the questionnaire was conducted to evaluate the clarity of the items, the participants’ comprehension, and the interviewers’ fieldwork skills (Escalante, 2026; García et al., 2026). Data collection took place between December 2023 and May 2024, with a team of interviewers who administered the instrument in person and explained the study’s objectives (Pomachagua et al., 2026). Furthermore, compliance with ethical standards, including anonymity and confidentiality, was ensured, and informed consent was obtained (Hernández-Sampieri and Mendoza, 2018). The average interview duration ranged from 25 to 30 min.

### 2.7 Research instrument

The study employed the Validated Organizational Climate Questionnaire (CCOV) developed by Bustamante and Álvarez (2019) for high-complexity hospitals in Chile and Ecuador. The instrument consists of 27 items grouped into four theoretical dimensions: (F1) Internal Management, conceptualized as an exogenous factor; (F2) Job Stability, as a mediating factor; (F3) Internal Tension and Professional Development, also as a mediating factor; and (F4) Organization and Performance, which acts as an endogenous factor. Perceptions were collected using a 5-point unidirectional Likert scale (Ramírez, 2026; Rangel-Baca and Pacheco-Paz, 2026). In accordance with this approach and with the aim of ensuring unidirectional scales in the analysis, items with negative wording were revised to their reverse form to guarantee a more accurate analysis. The proposed model was analyzed in two national segments, Chile and Ecuador, using a large combined sample (*N* = 1,222).

### 2.8 Exploratory factor analysis

Based on the stated objective of analyzing the organizational climate in two different realities using appropriate psychometric methods, an exploratory factor analysis (EFA) was chosen. Subsequently, given the capabilities of the method and previous evidence observed in this and other contexts (Bustamante et al., 2022; Rangel-Baca and Pacheco-Paz, 2026), the methodological guidelines proposed by Escalante (2026) were followed, in addition to the instrumental options provided by parallel analysis (PA) precisely to determine the latent variables (Hernandez and Diaz, 2026) applicable to a complex construct such as organizational climate (Ale, 2026; Morales et al., 2026). Consequently, and in order to address this complexity, the analysis was performed using the polychoric correlation matrix and employed robust unweighted least squares (ULS) estimation with direct oblimin rotation (Jiménez et al., 2026), since these methodological options allow for reducing the influence of outliers and the lack of normality (Hernández-Sampieri and Mendoza, 2018; Rodríguez and López, 2026).

Likewise, and based on the aforementioned, and considering the ordinal nature of Likert-type items, the use of polychoric correlations was justified and deemed pertinent (Ramírez, 2026), followed by verifying the relevance of the items using communality indices (≥ 0.5) and factor loadings (≥ 0.6) according to established criteria (Hernandez and Diaz, 2026; Shi et al., 2019). In this way, it is possible to ensure that the items adequately represent the underlying constructs (Escalante, 2026), in this case those of organizational climate, thus improving the interpretability of the resulting factor solution (Rangel-Baca and Pacheco-Paz, 2026).

Finally, the reliability of the instrument was verified using Cronbach’s alpha and McDonald’s omega (Antonio and Alberto, 2021) in such a way that, finally, the database was verified through the Kaiser-Meyer-Olkin (KMO) adequacy index (≥ 0.90) and Bartlett’s test of sphericity (Ramírez, 2026) considering a high explained variance (≥ 0.80) which is considered, according to various studies, an indicator of satisfactory factor structure adequacy (Secaira et al., 2026).

### 2.9 Confirmatory factor analysis and structural Modeling (SEM)

A confirmatory factor analysis (CFA) was performed to evaluate the factor structure obtained in the exploratory phase (Rojas-Torres, 2020). The analysis employed a polychoric correlation matrix (García et al., 2026) and a robust unweighted least squares estimation, which allows for the presence of latent correlated factors (Secaira et al., 2026).

Next, the model fit was evaluated using absolute fit indices (Domínguez-Lara, 2019), including the chi-square likelihood ratio (CMIN/DF ≤ 3) (García et al., 2026), the goodness of fit index (GFI ≥ 0.90) and the mean squared error of approximation (RMSEA ≤ 0.05 = good fit; ≤ 0.08 = acceptable fit) (Shi and Maydeu-Olivares, 2019). Incremental fit indices were also examined, including the comparative fit index (CFI), the adjusted goodness-of-fit index (AGFI) (≥ 0.9) (Cho et al., 2020), and the unnormalized fit index (Tucker-Lewis index, TLI), equivalent to the NNFI, with recommended values ≥ 0.90 (Xia and Yang, 2019). Additionally, 90% confidence intervals for the RMSEA were examined to assess the accuracy of the model fit (Jiménez et al., 2026; Shi et al., 2019).

### 2.10 Software use

Data analysis was performed using SPSS v.23 for descriptive and inferential analyses (Hernández-Sampieri and Mendoza, 2018). Exploratory factor analysis was conducted using Factor v.10.5.03, while confirmatory factor analysis and structural equation modeling were performed using SPSS AMOS v.21. G*Power 3.1 was used to calculate *a priori* and *post hoc* estimates (Secaira et al., 2026).

## 3 Results

The findings are presented in three stages: first, descriptive statistics of the samples; second, the results of inferential analyses performed using factor analysis; and finally, the structural model, which includes comparative analyses of the factors validated in both countries.

### 3.1 Description of the Chilean sample

The final sample size comprised 1,222 participants, exceeding the minimum estimated threshold (1,000). This total included 639 respondents from Chile and 583 from Ecuador.

As shown in Table 1, the majority of the Chilean sample (68.7%) was male. A slightly higher proportion of participants worked in public institutions (55.2%) than in private institutions (44.8%). Most respondents were between 26 and 43 years old (52.3%) and reported less than 4 years of service (51.1%). Regarding educational level, 48.5% held a professional degree, and in terms of marital status, married participants constituted the largest group (41.9%).

**Table 1:** Demographic Characteristics of the Chilean Sample.

Gender	*n*	(%)	Institution	*n*	(%)
Male	439	68.7	Public	353	55.2
Female	200	31.3	Private	286	44.8

Age	*n*	(%)	Years of service	*n*	(%)
Under 26 years	102	16.0	Under 4 years	329	51.1
26–34 years	195	30.5	4–10 years	144	22.5
35–43 years	139	21.8	11–16 years	29	4.5
44–52 years	113	17.7	17–22 years	88	13.8
53–61 years	69	10.3	23–30 years	18	2.8
Over 61 years	21	3.28	Over 30 years	31	4.9

Educational level	*n*	(%)	Marital status	*n*	(%)
Technical degree	128	20.0	Single	231	36.2
Professional degree	310	48.5	Married	268	41.9
Master’s degree	56	8.8	Divorced	70	11.0
Doctorate (PhD)	10	1.6	Cohabiting	64	10.0
Specialization	134	21.0	Widowed	6	0.9

Profession	*n*	(%)	Contract type	*n*	(%)
Physician	304	47.6	Permanent appointment	378	59.2
Registered nurse	184	28.8	Temporary contract	208	32.6
Nursing assistant	151	23.6	Professional services	52	08.1

Hospital	*n*	(%)	Hospital complexity	*n*	(%)
Hospital 1	223	34.9	Medium complexity	13	2.0
Hospital 2	137	21.4	High complexity	626	98.0
Hospital 3	99	15.5			
Hospital 4	180	28.2			

n = Frequency; % = Percentage.

### 3.2 Description of the Ecuadorian sample

Table 2 details the results for the Ecuadorian sample, where the male segment was predominant (69.5%).

**Table 2:** Demographic characteristics of the Ecuadorian sample.

Gender	*n*	(%)	Institution	*n*	(%)
Male	405	69.5	Public	353	60.5
Female	178	30.5	Private	230	39.5

Age	*n*	(%)	Years of service	*n*	(%)
Under 26 years	98	16.8	Under 4 years	294	50.4
26–34 years	169	29.0	4–10 years	137	23.5
35–43 years	131	22.5	11–16 years	28	4.8
44–52 years	105	18.0	17–22 years	82	14.1
53–61 years	62	10.6	23–30 years	18	3.1
Over 61 years	18	3.1	Over 30 years	24	4.1

Educational level	*n*	(%)	Marital status	*N*	(%)
Technical degree	120	20.6	Single	213	36.5
Professional degree	280	48.0	Married	244	41.9
Master’s degree	54	9.3	Divorced	61	10.5
Doctorate (PhD)	9	1.5	Cohabiting	60	10.3
Specialization	119	20.4	Widowed	5	0.9

Profession	*n*	(%)	Contract type	*N*	(%)
Physician	268	46.0	Permanent appointment	342	58.7
Registered nurse	173	29.7	Temporary contract	199	34.1
Nursing assistant	142	24.4	Professional services	41	7.0

Hospital	*n*	(%)	Hospital complexity	*N*	(%)
Hospital 1	223	38.3	Medium complexity	12	2.1
Hospital 2	137	23.5	High complexity	571	97.9
Hospital 3	94	16.1			
Hospital 4	129	22.1			

n = Frequency; % = Percentage.

Regarding institutional affiliation, 60.5% of respondents were public sector employees. The majority of participants were between 26 and 43 years old (51.5%) and had less than 4 years of service (50.4%). In terms of education level, the largest proportion held a professional degree (48.0%), and regarding marital status, the majority of respondents were married (41.9%).

The variables of the Validated Organizational Climate Questionnaire developed by Bustamante and Álvarez (2019) showed similar patterns in both Chile and Ecuador, with mean scores above 3 on a 5-point Likert scale.

Ecuador presented slightly higher overall mean scores than Chile (3.431 vs. 3.406), as well as higher standard deviations (1.149 vs. 1.124) and variances (1.325 vs. 1.269).

### 3.3 Descriptive analysis of organizational climate variables

Descriptive statistics for the 27 organizational climate items analyzed are presented in Table 3.

**Table 3:** Descriptive analysis of organizational climate variables in Chile and Ecuador.

Organizational climate model variables (27 items)	Chile			Ecuador		
	μ	𝝈	𝝈2	μ	𝝈	𝝈2
When I have to do a difficult task, I can count on the help of my colleagues.	3.91	1.09	1.20	3.95	1.06	1.12
Managers provide feedback to their staff regarding their job performance.	3.75	1.08	1.18	3.79	1.06	1.13
Employees know what their supervisors expect of them.	3.87	1.14	1.29	3.92	1.09	1.19
The managers promote good interpersonal relationships among the people in the institution.	3.70	1.12	1.27	3.75	1.09	1.19
The managers in this institution treat their subordinates with respect.	3.79	1.12	1.27	3.83	1.09	1.20
When I have to do a difficult task, I can count on the help of my manager(s).	3.78	1.12	1.27	3.81	1.10	1.22
In this institution, people are committed to their work.	3.84	1.06	1.14	3.90	1.02	1.04
The people in this institution make a great effort to perform their work efficiently.	3.86	1.04	1.08	3.91	0.99	0.99
The people in this institution show interest in the work they do.	3.86	1.06	1.13	3.92	1.02	1.05
Here, it is easy for any employee to present a new idea.	3.44	1.09	1.20	3.47	1.07	1.15
I’m worried that a reorganization at the institution could affect my job security.	3.44	1.24	1.53	3.45	1.22	1.48
I’m concerned about the potential impact of changes in work methods and automation at this institution on my job security.	3.38	1.18	1.41	3.40	1.15	1.33
I believe I could lose my job at this institution at any moment.	3.33	1.26	1.61	3.33	1.23	1.52
This institution does not provide opportunities to develop personal skills and abilities.	3.04	1.13	1.27	3.04	1.12	1.26
Employees at this institution tend to trust rumors about certain events more than official information.	3.12	1.21	1.46	3.11	1.21	1.47
This institution is characterized by a tense work environment.	3.11	1.20	1.45	3.11	1.20	1.44
At this institution, we are kept uninformed about matters we should know.	2.89	1.21	1.46	2.88	1.20	1.45
The atmosphere at this institution is not conducive to developing new ideas.	2.91	1.13	1.28	2.91	1.12	1.25
New ideas contributed by staff are not well received by management.	2.90	1.10	1.21	2.91	1.08	1.17
There is too much criticism at this institution.	3.08	1.22	1.50	3.07	1.21	1.46
Sometimes we work in a disorganized and unplanned manner.	2.95	1.28	1.64	2.95	1.27	1.63
The management of this institution cares about people, how they feel, and their problems.	3.13	1.17	1.37	3.18	1.16	1.35
In this institution, tasks are well assigned and organized.	3.35	1.17	1.37	3.41	1.12	1.25
Ability is the primary criterion for assigning tasks in this institution.	3.44	1.07	1.14	3.48	1.02	1.04
There is good communication between management and staff.	3.17	1.20	1.44	3.21	1.18	1.39
Everything that needs to be done is clear because it is explained to us well and in a timely manner.	3.28	1.15	1.32	3.32	1.12	1.25
There is good communication between the different units and services, which generally work together.	3.33	1.14	1.30	3.36	1.12	1.25
Overall means	3.28	1.15	1.32	3.33	1.12	1,26

μ = Mean; 𝝈 = Standard Deviation; 𝝈𝟐 = Variance.

The first section of the instrument describes how daily activities are managed within the organization, explicitly detailing task assignment, instructions, and respectful treatment, similar to how job satisfaction has been studied in other jobs of a similar nature (Nieto et al., 2026). This fosters a sense of commitment and motivates employee effort.

Next, in the second part, the analyzed items refer to components related to the risk of job insecurity and the impact of process automation, the management of which affects the organizational climate through structural and organizational components (Rodríguez and López, 2026).

And, the third section of the instrument describes a set of variables that behave independently within the organizational climate model and, from that perspective, influence the perceptions of healthcare workers (Angeles-Cabrera et al., 2026) depending on the environment, information networks, and active listening, which act as drivers of internal behavior (Ale, 2026).

Finally, the fourth factor of organizational climate can be interpreted as the set of effects that the management of the elements produces in the perception of the workers (Alvear, 2026), who appreciate the tasks that are assigned to them, the related information and the well-being generated by the connections established between people through their respective units, which improves the productivity of the organizations (Vasquez et al., 2026).

### 3.4 Inferential analysis of the organizational climate construct

In the inferential phase, an exploratory factor analysis was conducted on the 27 organizational climate items (Table 3) using the Hot Deck multiple imputation method to handle missing data (Lorenzo-Seva and Fernando, 2013). Factor extraction was guided by parallel analysis (PA) using a Pearson correlation matrix (Shi et al., 2019).

Sampling adequacy was assessed using the Kaiser–Meyer–Olkin (KMO) index. The Chilean sample (*n* = 639) and Ecuadorian (*n* = 583) samples yielded KMO values of 0.91 and 0.910, respectively, both exceeding the stringent threshold of 0.90, indicating excellent suitability for factor analysis.

Internal consistency reliability was assessed using Cronbach’s alpha and McDonald’s omega. In the Chilean sample, Cronbach’s alpha was 0.90, and McDonald’s omega was 0.755. In the Ecuadorian sample, Cronbach’s alpha was 0.87, and McDonald’s omega was 0.825, supporting the reliability of the extracted factor structures (Xia and Yang, 2019).

Finally, the Comparative Fit Index (CFI) was calculated as a supplementary indicator of model adequacy, yielding values of 0.91 for Chile and 0.90 for Ecuador. Table 4 presents the results of the exploratory factor analysis.

**Table 4:** Results of the exploratory factor analysis of organizational climate.

**In Chilean high-complexity hospitals**		FL	Com.
C	Factor 1: management support and work environment		
V14	The managers promote good interpersonal relationships among the people in the institution.	0.71	0.82
V16	When I have to do a difficult task, I can count on the help of my manager(s).	0.72	0.85
V 30	This institution is characterized by a tense work environment.	0.74	0.82
V 35	There is a high level of respect for people in this institution.	0.81	0.72
V 36	People in this institution do not trust each other.	0.85	0.87
V 37	Sometimes we work in a disorganized and unplanned way.	0.76	0.70
VAR	Factor 2: tensions and conflicts		
V 26	The managers believe that disagreements between different units and individuals can be beneficial for the institution’s improvement.	0.78	0.76
V 27	There are very few opportunities for advancement in this institution.	0.84	0.83
V 28	This institution does not provide opportunities to develop personal skills and abilities.	0.70	0.67
VAR	Factor 3: support for intrapreneurship		
V 32	The atmosphere in this institution is not conducive to developing new ideas.	0.77	0.77
V 33	New ideas contributed by staff are not well received by management.	0.81	0.84
V 34	There is too much criticism in this institution.	0.66	0.74

EV	F1 = 34.5**	F2 = 19.7**	F3 = 7.0*	Total = 61.2	Cronbach’s α	0.90
KMO	0.91	CFI	0.91		McDonald’s ω	0.75

In Ecuadorian high-complexity hospitals		Factor loading	Communality
VAR	Factor 1: collaboration, respectful treatment, support, and trust		
V 14	The managers promote good interpersonal relationships among the people in the institution.	0.72	0.80
V 16	When I have to do a difficult task, I can count on the help of my manager(s).	0.74	0.81
V 20	The people in this institution show interest in the work they do.	0.71	0.76
V 30	This institution is characterized by a tense work environment.	0.76	0.91
V 35	There is a high level of respect for people in this institution.	0.80	0.76
V 36	People in this institution do not trust each other.	0.83	0.80
V 37	Sometimes we work in a disorganized and unplanned way.	0.76	0.72
VAR	Factor 2: relationships, friendship, and growth		
V 24	In this institution, a friendly atmosphere prevails among the staff.	0.71	0.83
V 26	The managers believe that disagreements between different units and individuals can be beneficial for the institution’s improvement.	0.77	0.72
V 27	There are very few opportunities for advancement in this institution.	0.82	0.86
VAR	Factor 3: support for intrapreneurship		
V 32	The atmosphere in this institution is not conducive to developing new ideas.	0.81	0.84
V 33	New ideas contributed by staff are not well received by management.	0.80	0.85
V 34	There is too much criticism in this institution.	0.59	0.73

EV	F1 = 33.7**	F2 = 20.3**	F3 = 6.9*	Total = 60.9	Cronbach’s α	0.87
KMO	0.91	CFI	0.90		McDonald’s ω	0.82

C = Code; FL = Factor loading; EV = Explained Variance; Com. = Communality.

### 3.5 Organizational climate in the Chilean healthcare context

The results identified three distinct factors comprising 12 of the 27 analyzed items, which explained 61.2% of the total variance.

The first factor (F1), Management Support and Work Environment, consisted of six items and accounted for 34.5% of the total variance. It represents the degree to which employees perceive management as supportive, particularly in terms of interpersonal collaboration and conflict reduction. While this factor signals a foundation of mutual trust, structural disorganization and poor task planning remain a source of employee uncertainty.

The second factor (F2), Tensions and Conflicts, included three items and explained 19.7% of the variance. This factor captures perceptions of how discrepancies between units and individuals are managed, with a view toward institutional improvement. However, it also highlights that limited opportunities for promotion constrain professional development.

The third factor (F3), Support for Intrapreneurship, explained 7.0% of the variance and consisted of three items. This factor identifies an environment perceived as unfavorable to new ideas, as they are poorly received by management and met with excessive criticism, which in turn contributes to workplace tension.

### 3.6 Organizational climate in the Ecuadorian healthcare context

In the Ecuadorian context, the analysis of the 27-item instrument yielded 13 items, grouped into three factors, which accounted for 60.9% of the total variance.

The first factor (F1), Collaboration, Respectful Treatment, Support, and Trust, comprised seven items and explained 33.7% of the variance. It describes how managerial practices promote positive interpersonal relationships and support during demanding situations. While respect for individuals is high, trust remains perceived as limited, and work processes are often seen as disorganized.

The second factor (F2), Relationships, Friendship, and Growth, comprised three items and accounted for 20.3% of the variance. It reflects a friendly work environment in which disagreements are viewed as opportunities for growth, though professional advancement remains restricted.

The third factor (F3), Support for Intrapreneurship, included three items and explained 6.9% of the variance. This factor captures perceptions of resistance to new ideas and the situations in which managerial criticism generates significant tension.

Having described the factors identified in this phase of the analysis, it is worth mentioning that of the 27 items studied, the factor modeling, meeting the requirements of the methodology (Ale, 2026; Morales et al., 2026), only allowed for the identification of three factors duly grounded in both contexts. This confirmed the participation of 12 items in the factor model of organizational climate in hospitals in Chile and 13 variables in the hospital factor model in Ecuador. Consequently, in both studies, approximately 44% of the total variables analyzed were excluded due to insufficient factor loadings and communalities (Hernandez and Diaz, 2026; Shi et al., 2019) or because the required number of items to form factors was not adequately met (Rangel-Baca and Pacheco-Paz, 2026).

### 3.7 Structural covariance modeling of organizational climate

**Figure 1:**
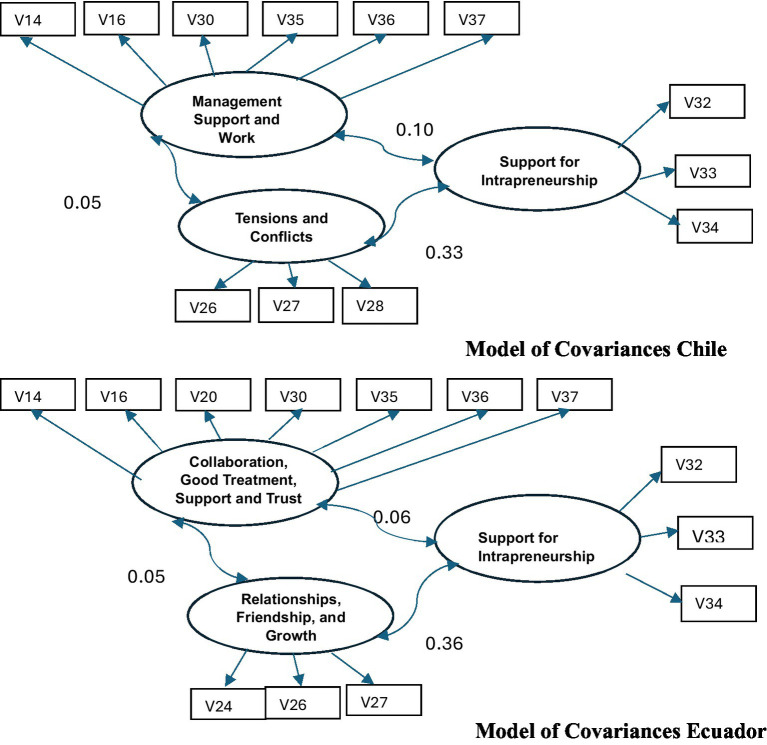
Structural covariance modeling of organizational climate.

As illustrated in Figures 1, a covariance analysis was performed to assess the significance of correlations among factors. In both countries, the coefficients fell within the expected range (0–1), indicating that these dimensions are reliable for management decision-making.

In Chile, Management Support and Work Environment was positively related to Support for Intrapreneurship (*r* = 0.05) and Tensions and Conflicts (*r* = 0.10). In addition, Support for Intrapreneurship showed a direct association with Tensions and Conflicts (*r* = 0.33). This indicates that perceptions of intrapreneurship are associated with both supportive and conflict-related dimensions of organizational climate.

In Ecuador, Collaboration, Respectful Treatment, Support, and Trust was directly and positively related to Support for Intrapreneurship (*r* = 0.06). Support for Intrapreneurship was associated with Relationships, Friendship, and Growth (*r* = 0.36), which in turn was related back to the first factor (*r* = 0.05). These results confirm a multidimensional structure that provides a useful framework for improving the organizational climate in Ecuadorian healthcare.

Complementing the analysis and interpretation of the factors under study, it is possible to affirm that, both in the Chilean and Ecuadorian contexts, without this demonstrating a transcultural adequacy of the findings, the first factor (F1) Collaboration, respectful treatment and support, arises from the more appropriate exercise of soft skills by supervisors and managers (Ale, 2026; Lai, 2020), certainly in different magnitudes but in equivalent relationships, since it derives from executive practices that influence the staff and impact the second factor (F2) Relationships, friendship and growth, generating virtuous effects, as occurs with staff turnover (Alvear, 2026) and, progressively, in general performance, as has been demonstrated in systematic reviews of the organizational climate (Angeles-Cabrera et al., 2026).

Finally, factor F1 is directly related to factor (F3) Support for intrapreneurship, progressively affecting the various organizational dimensions, as has been demonstrated in several industrial contexts (Morales et al., 2026; Vasquez et al., 2026; Xia and Yang, 2019), and that this work affirms that they are established, with caution in cross-cultural terms in the absence of additional statistical evidence, in the health sector of high-complexity hospitals.

### 3.8 Structural variance modeling of organizational climate

**Figure 2:**
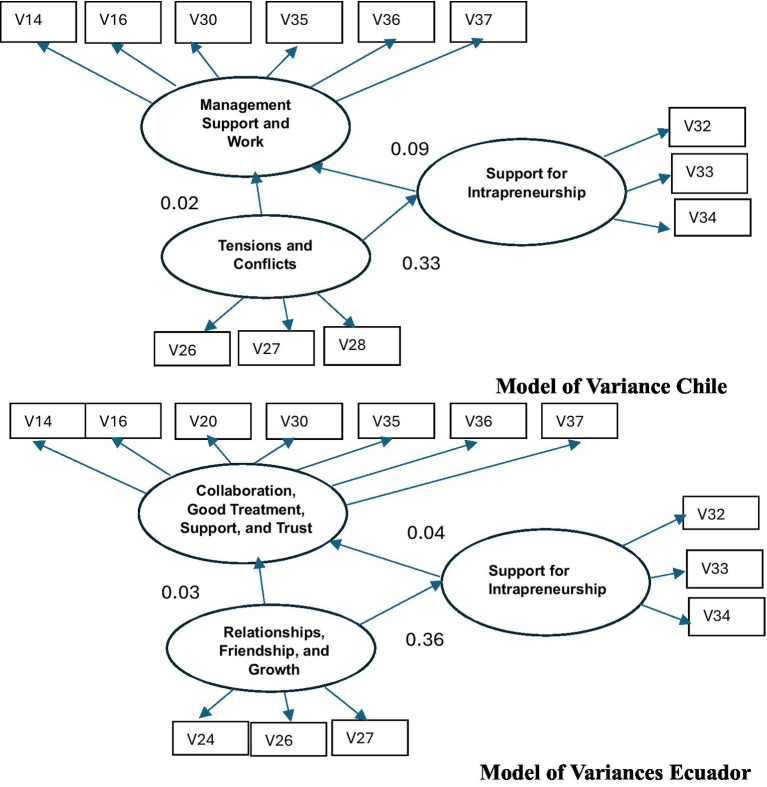
Structural variance modeling of organizational climate.

Figure 2 presents the structural variance model obtained in the second stage of analysis. This phase examined directional relationships among the latent factors.

In the Chilean context, Management Support and Work Environment served as the endogenous factor, receiving a direct and positive influence from Support for Intrapreneurship (*β* = 0.09) and, to a lesser extent, from Tensions and Conflicts (*β* = 0.02). At the same time, Tensions and Conflicts, as an exogenous factor, exerted a direct and significant influence on Support for Intrapreneurship (*β* = 0.33), which serves as a mediating dimension within the proposed model.

The results indicate that, in Chile, organizational climate was structured through sequential relationships, reflecting healthcare workers’ perceptions of their organizational reality.

In the Ecuadorian model, Relationships, Friendship, and Growth operated as the exogenous latent factor. This factor exerted a direct influence on the endogenous factor Collaboration, Respectful Treatment, Support, and Trust (*β* = 0.03).

In turn, Collaboration, Respectful Treatment, Support, and Trust showed a strong direct influence on Support for Intrapreneurship, which acted as the mediating factor (*β* = 0.36). Finally, Support for Intrapreneurship exerted a smaller direct effect on the endogenous factor (*β* = 0.04), confirming the presence of sequential relationships among the dimensions.

The variance modeling results show that the organizational climate perceptions in Ecuadorian High-complexity hospitals are characterized by interconnected relationships among exogenous, mediating, and endogenous factors.

As shown by the incidence indices in Figure 2, for the two contexts studied in Chile and Ecuador, the three factors under analysis establish impact relationships, beginning with factor (F2) Relationships, friendship, and growth, which directly influences factor (F1) Collaboration, respectful treatment, and support. This factor, in turn, is influenced by the third factor (F3) Support for intrapreneurship, as evidenced by standardized indices shown within equivalent magnitude ranges.

This initial impact allows us to conclude that factor F1 assumes the role of an effect factor, conditioned by the impacts of factors F2 and F3 (Lai, 2020; Xia and Yang, 2019). These latter two factors are positioned as causal factors that, individually or in combination, as has already been studied (Nieto et al., 2026), can reduce or potentially increase job satisfaction in healthcare workplaces.

Furthermore, it is also possible to state that the third factor (F3) Support for intrapreneurship, identified by Miguel in both hospital realities of Chile and Ecuador, cannot be confirmed at a transcultural and generalizable level; however, in both realities it is impacted by the second factor (F2), and is located in the role of a mediating factor since through it, factor (F2) indirectly affects factor (F1), shaping an articulated network of factors that explain the organizational climate, demonstrating an interesting coincidence that should be highlighted, although with the necessary caution, although, as has been shown in other contexts, it affects job performance (Murillo Núñez, 2026) and as already mentioned, mutual factorial relationships are established in this work carried out in high complexity hospitals in Chile and Ecuador.

### 3.9 Goodness-of-fit estimates for organizational climate factors in Chile and Ecuador

The goodness-of-fit indices for the covariance and variance models are shown in Table 5. In both contexts, the CMIN/DF index remained below the recommended threshold (≤3), indicating an acceptable model fit.

**Table 5:** Goodness-of-fit indices for covariance and variance models in Chile and Ecuador.

**Covariance model fit indices–Chile**					
**Model**	NPAR	CMIN	DF	P	CMIN/DF
Default model	41	108.01	49	0.00	**2,204**

**Baseline comparisons**	NFI	RFI	IFI	TLI	CFI
Default model	**0.97**	**0.96**	**0.98**	**0.97**	**0.98**

**RMSEA**	RMSEA	LO 90	HI 90	PCLOSE
Default model	**0.04**	0.03	0.05	0.82
Independence model	0.30	0.29	0.30	0.00

**Covariance model fit indices Ecuador**					
**Model**	NPAR	CMIN	DF	P	CMIN/DF
Default model	46	153.58	58	0.00	**2.64**

**Baseline comparisons**	NFI	RFI	IFI	TLI	CFI
Default model	**0.96**	**0.94**	**0.97**	**0.96**	**0.97**

**RMSEA**	**RMSEA**	LO 90	HI 90	PCLOSE
Default model	**0.05**	0.04	0.06	0.28

**Variance model fit indices–Chile**					
**Model**	NPAR	CMIN	DF	P	**CMIN/DF**
Default model	41	108.01	49	0.00	**2.20**

**Baseline comparisons**	NFI	RFI	IFI	TLI	CFI
Default model	**0.97**	**0.96**	**0.98**	**0.97**	**0.98**

**RMSEA**	**RMSEA**	LO 90	HI 90	PCLOSE
Default model	**0.04**	0.03	0.05	0.82

**Variance model fit indices–Ecuador**					
	NPAR	CMIN	DF	P	**CMIN/DF**
Default model	46	153.58	58	0.00	**2.64**

**Baseline comparisons**	NFI	RFI	IFI	TLI	CFI
Default model	**0.96**	**0.94**	**0.97**	**0.96**	**0.97**

**RMSEA**	**RMSEA**	LO 90	HI 90	PCLOSE
Default model	**0.05**	0.04	0.06	0.28

NPAR (Number of parameters); CMIN (Statistic (chi2)); DF (Degrees of freedom); P (Significance level of the CMIN); NFI (Normed Fit Index); RFI (Relative Fit Index); IFI (Incremental Fit Index); TLI (Tucker-Lewis Index); CFI (Comparative Fit Index); RMSEA: Mean Squared Error of Approximation; LO 90: It is the lower limit; HI 90: Upper limit; PCLOSE: Close FitFor NFI, RFI, IFI, TLI, and CFI = Values in italics and bold are above the minimum acceptable fit criterion (0.90); For RMSEA = Values are below the maximum required (0.05), confirming the fit; The estimators in bold confirm the obtained model.

For the covariance models, the CMIN/DF values were 2.20 for Chile and 2.64 for Ecuador. Similarly, the variance (incidence) models yielded CMIN/DF values below the acceptable limit for both countries, further supporting the adequacy of the proposed models.

Incremental and comparative fit indices exceeded recommended cutoff values. In the Chilean model, the indices reached NFI = 0.97, RFI = 0.96, IFI = 0.98, TLI = 0.97, and CFI = 0.98. Likewise, the Ecuadorian model showed satisfactory fit indices (NFI = 0.96, RFI = 0.94, IFI = 0.97, TLI = 0.96, and CFI = 0.97), confirming good fit in both contexts.

The root mean square error of approximation (RMSEA) further supported these results. The RMSEA value for the Chilean model was 0.04, indicating close fit, whereas the Ecuadorian model yielded an RMSEA of 0.05, indicating acceptable fit. In both cases, RMSEA values fell within the recommended confidence intervals for adequate model fit (Hernandez and Diaz, 2026; Lai, 2020).

### 3.10 Confirmatory structural equation model of organizational climate factors

Table 6 shows the standardized *β* coefficients for the structural equations, estimating the effects of the exogenous and mediating factors on the endogenous factor in both contexts. All reported structural coefficients were statistically significant at the levels indicated in Table 6.

**Table 6:** Structural equations of organizational climate factors.

**Structural equations–Chile**	
F3 = 0.44*** F2 + 0.98***	(1)
F1 = 0.05* F3 + 0.02 F2 + 0.43***	(2)

**Structural equations–Ecuador**	
F3 = 0.39*** F2 + 0.97***	(3)
F1 = 0.02 F3 + 0.02 F2 + 0.39***	(4)

In the Chilean context, equation (1) shows the directional relationship (F3 < --- F2) and the error coefficients for the mediating variable Tensions and Conflicts, which has a significant effect on the factor Management Support and Work Environment.

Equation (2) illustrates the sequential effects (F1 < --- F3 and F1 < --- F2), demonstrating that the exogenous factor Tensions and Conflicts had a direct effect on the endogenous factor Management Support and Work Environment, as well as an indirect effect through the mediating factor Intrapreneurship Support.

In the Ecuadorian context, equation (3) specifies the directional relationship (F3 < --- F2) for the exogenous factor Relationships, Friendship, and Growth, which influenced the endogenous factor Collaboration, Respectful Treatment, Support, and Trust.

Finally, equation (4) describes the directional relationships (F1 < --- F3 and F1 < --- F2) of the exogenous factor Relationships, Friendship, and Growth, which directly affected the endogenous factor Collaboration, Respectful Treatment, Support, and Trust, while mediating factor Support for Intrapreneurship also exerted an effect on this endogenous factor.

## 4 Discussion

Factor analysis of the Chilean data identified three latent factors: (F1) Management Support and Work Environment, associated with perceptions of managerial support and organizational commitment to fostering an environment of respect and mutual trust; (F2) Tensions and Conflicts, referring to disagreements between different units and individuals linked to limited growth opportunities; and (F3) Support for Intrapreneurship, reflecting perceptions of how innovative ideas are received within the organization. These findings are consistent with previous research on an ethical organizational climate that recognizes the importance of self-reflection and self-awareness in decision-making (Carpes Lanes et al., 2024) and provide relevant information for developing interventions that strengthen the work environment in the Chilean context, as has been demonstrated (Barría-González et al., 2025).

In the Ecuadorian case, factor (F1), Collaboration, Respectful Treatment, Support, and Trust, reflects employees’ valuation of work activities, managerial support, and trust-based relationships. Complementarily, factor (F2), Relationships, Friendship, and Growth, reflects perceptions of friendly interpersonal relationships that foster growth opportunities despite disagreements. Finally, (F3), Support for Intrapreneurship, reflects how organizations address new ideas in contexts where criticism and tension can affect performance. Together, these dimensions form an organizational climate construct closely related to organizational culture, highlighting how management impacts areas of the work climate and creating significant opportunities to improve coexistence and the physical work environment (Alfredo et al., 2025; Bustamante et al., 2022).

Factorial relationships in Chilean hospital institutions showed the existence of exogenous factors Support for Intrapreneurship and Tensions and Conflicts that influence the endogenous factor Management Support and Work Environment, where the factorial dimension Support for Intrapreneurship, in turn acts as a mediating variable, influenced by the Tensions and Conflicts factor, establishing a pattern of relationships (Angeles-Cabrera et al., 2026) that highlights how organizational values, beliefs and practices shape perceptions of the organizational climate (Hernandez and Diaz, 2026).

In Ecuador, the exogenous factor of Relationships, Friendship, and Growth, along with Support for Intrapreneurship, influences the endogenous dimension of Collaboration, Respectful Treatment, Support, and Trust. In turn, the mediating factor, Support for Intrapreneurship, was influenced by Relationships, Friendship, and Growth, forming a sequence of interrelated soft skills (Ale, 2026). These findings align with those of Terán et al. (2024), who concluded that positive working conditions are associated with higher levels of employee productivity. In this sense, the modeled relationships suggest that the dimensions of organizational climate can influence work outcomes (Morales et al., 2026).

Furthermore, structural equation modeling, as a broad generic model in this type of study (Rodríguez and López, 2026), allows us to confirm in the Chilean context that the exogenous variable Tensions and Conflicts influences the endogenous factor Management Support and Work Environment (Bustamante et al., 2022). Likewise, both Tensions and Conflicts and Intrapreneurial Support influence this endogenous factor. These findings support the proposed organizational climate model, which emphasizes the influence of conflict dynamics and intrapreneurial support on managerial support and labor productivity, as demonstrated in other industrial sectors (Vasquez et al., 2026). This also aligns with the findings of Doria-Velarde et al. (2023) in the Maule region of Chile, who reported associations between favorable work climates and skills development, thereby increasing job satisfaction (Nieto et al., 2026).

In the Ecuadorian healthcare context, the exogenous factor Relationships, Friendship, and Growth influenced the endogenous factor Collaboration, Respectful Treatment, Support, and Trust. Furthermore, both the exogenous factor Relationships, Friendship, and Growth and the mediating factor Support for Intrapreneurship influenced the same dependent factor, resulting in an organizational climate model adapted to the Ecuadorian context (Javier et al., 2025). Additionally, as Guevara-Rufasto and Peñalver-Higuera (2024) point out, these models depend on employees’ perceptions and interactions with organizational elements, including policies, methods, and culture experienced by healthcare institutions in the long term, which in turn stems from the perception of the organization’s climate in the short term (Carpes Lanes et al., 2024).

The similarities observed between both countries suggest that interpersonal support and managerial treatment constitute central dimensions of organizational climate in Latin American healthcare institutions. However, the emergence of tensions and conflicts as an exogenous factor in Chile, compared with relationships and friendship in Ecuador, may reflect contextual differences in organizational dynamics, institutional culture, and social interaction patterns.

## 5 Conclusion

This study psychometrically validated the organizational climate construct in high-complexity hospitals in Chile and Ecuador, confirming the existence of three-factor structures with satisfactory reliability and goodness-of-fit indicators in both contexts.

In Chile, the organizational climate construct was composed of the factors: (F1) Management Support and Work Environment, (F2) Tensions and Conflicts, and (F3) Support for Intrapreneurship, explaining 61.2% of the total variance. In Ecuador, the identified factors were: (F1) Collaboration, Respectful Treatment, Support, and Trust, (F2) Relationships, Friendship, and Growth, and (F3) Support for Intrapreneurship, explaining 60.9% of the total variance.

The results revealed differences in the exogenous dimensions identified in each context. In Chile, Tensions and Conflicts emerged as the active exogenous factor, whereas in Ecuador the predominant exogenous dimension was Relationships, Friendship, and Growth, reflecting contextual differences in organizational dynamics and interpersonal relationships within healthcare institutions.

Despite these differences, both contexts showed similar endogenous and mediating structures associated with managerial support, interpersonal collaboration, and intrapreneurship support. Notably, the factor Support for Intrapreneurship was identified with the same items in both countries, suggesting partial consistency in the organizational climate construct across the analyzed healthcare environments, although broader cross-cultural generalization cannot yet be confirmed.

Overall, the findings demonstrate that organizational climate in high-complexity hospitals is shaped by interconnected factorial dimensions influenced by local organizational and cultural conditions. These results provide relevant evidence for healthcare management and open important opportunities for future comparative research in primary healthcare systems and other Latin American institutional contexts.

### 5.1 Limitations and suggestions

This study has some limitations that should be acknowledged. Data collection was conducted in two different national contexts, which may influence participants’ interpretations of the instrument items despite the pilot testing procedures carried out prior to data collection. In addition, organizational climate perceptions may contain subjective and emotional components inherent to self-report instruments.

To reduce these effects, equivalent psychometric and analytical procedures were applied in both countries to ensure comparability between contexts. Future studies could expand the analysis to primary healthcare institutions and other Latin American healthcare systems in order to further evaluate the cross-cultural stability of the organizational climate construct.

## Data Availability

The raw data supporting the conclusions of this article will be made available by the authors, without undue reservation.
